# The Validity and Reliability of Malay Version Child Exposure to Domestic Violence Scale

**DOI:** 10.21315/mjms2023.30.4.15

**Published:** 2023-08-24

**Authors:** Tina Azreen Norazman, Surini Yusoff, Hanif Mohd Niza@Nizal, Fahisham Taib

**Affiliations:** 1 Department of Pediatric, School of Medical Sciences, Universiti Sains Malaysia, Kelantan, Malaysia; 2 Department of Paediatric, Hospital Universiti Sains Malaysia, Kelantan, Malaysia; 3 Department of Paediatric, Hospital Tuanku Fauziah, Perlis, Malaysia

**Keywords:** maltreatment, Child Exposure to Domestic Violence, child abuse, validity, reliability

## Abstract

**Background:**

Domestic violence has a significant impact on growing children. However, existing evidence is limited and often under reported. Consequently, the Child Exposure to Domestic Violence (CEDV) scale has been developed for global use. This study aims to provide a cross-cultural translation, adaptation and validation of the CEDV based on Malay language.

**Methods:**

CEDV scale was translated from the original English version to Malay. Content and face validity were examined before field-testing. The study comprised a cross sectional study using the Malay version of the CEDV (CEDV-M) scale conducted at a secondary school in Perlis, Malaysia and investigated 235 children aged 13 years old–16 years old using an online platform. The construct validity, internal consistency and stability of the tool were assessed.

**Results:**

The CEDV-M scale’s content and face validity both yielded a value exceeding 0.80. Furthermore, the tool demonstrated has good stability reliability, using the intra-class correlation value for all items ranging from 0.659 to 1.00. The exploratory factor analysis of the 36 items of the CEDV scale revealed possibilities of five or six factor domains. However, the five factor domains were most conceptually equivalent. Consequently, this study found that the CEDV-M scale is reliable with a total Cronbach’s alpha of 0.87.

**Conclusion:**

CEDV-M scale is a valid and reliable tool for measuring a child’s experience of domestic violence. Future studies recommend confirmatory factor analysis and standard settings for scoring systems.

## Introduction

Domestic violence is described as violence occurring among intimate partners who are either spouses or ex-spouse of a legal relationship, as stated in Malaysia’s Domestic Violence Act 521 (1994). The impact of violence is usually followed by consequences, for the victims as well as for their children, families, and surrounding communities. Studies have found that children exposed to domestic violence or living in an abusive relationship may also experience direct abuse (1, 2) and develop behavioural issues that may affect their emotional and social functioning (3). Furthermore, these individuals are likely to exhibit attitude problems, academic impairment and temperament issues (4).

Globally, approximately 1 billion children aged 2 years old–17 years old have experienced physical, sexual or emotional violence or neglect, with Asians comprising the regions with the highest minimum prevalence (5). In Malaysia, there is limited data and the prevalence of domestic violence and maltreatment among children is most likely under-reported, as it is considered a sensitive issue among communities. The available data are only from reported cases compiled by the Royal Malaysian Police, the Welfare Department and the Ministry of Health Malaysia. The latest published 2019 Social Welfare Statistics Reports, reported 673 reported cases of domestic violence throughout the country and found that a total of 6,382 children required protective care under Section 17 of the Child Act 2001. In 2014, large scale survey conducted in Malaysia was significant result as 75% of children aged 10 years old–12 years old in Selangor experienced at least one form of child maltreatment (6).

Violence against children can be prevented. Accordingly, the 2030 Agenda for Sustainable Development aims to end abuse, exploitation, trafficking and all forms of violence against children. A study has shown that a child-centred approach based on a self-reported tool is proven can elicit more instances of violence and yield more accurate reports than official police reports and child protective services statistics (7). Currently, several questionnaires that utilise children’s self-report of exposure to domestic violence are available. Notably, most of the existing questionnaires measure the impact of exposure to violence. However, the questionnaires cannot examine direct individual experiences with violence that could potentially affect people’s reaction to violent contexts (8). Therefore, a comprehensive assessment is needed considering the nature and complexity of children’s exposure to domestic violence (9). In 2008, Edleson et al. (8) developed the Child Exposure to Domestic Violence (CEDV) scale to address the lack of measures specific to children’s experience with domestic violence.

Even though the national language in Malaysia is Malay, the self-reported and universal assessment tools for measurement have limited availability in the country. Thus, this study aimed to provide a cross-cultural translation of a validated assessment tool in Malay language and to assess the level of exposure to domestic violence from a child’s perspective.

## Methods

### CEDV Scale

The CEDV scale is a self-administered assessment tool designed for children aged 10 years old–16 years old. The scale consists of 42 items, divided into three parts. Part I and Part II of the CEDV scale contains six subscales, that measure: i) level of violence in the home; ii) level of exposure to home violence; iii) level of exposure to community violence; iv) level of involvement in violent events; v) risk factors in home life; and vi) other victimisations experienced by the child at home. Each subscale is represented by 4 to 10 items. Part I had a total of 10 items with two sections each. The first section specifically targeted the types or the level of home violence (subscale 1). Here, each child was asked to rate the items using a 4-point Likert-type scale (never = 0, sometimes = 1, often = 2 and almost always = 3). The second section of Part I required the child to indicate how they knew about home violence (subscale 2). The child could proceed to the next item upon responding ‘Never’ to the item asked. However, if the child indicated exposure to violence, the child would be led to an additional set of options to choose to further on the exposure.

Part II of the CEDV scale includes 23 items, which were also answered using a similar 4-point Likert-type scale. These sections examined community violence exposure (items 22–29), involvement in violence (items 11–17), risk factors (items 18–21) and other types of victimisations experienced by the child (items 30–33). Subsequently, the response values for all items within a subscale were added. Higher scores indicate more violence, while lower scores indicate less violence, exposure, involvement, risk factors or other forms of victimisation depending on the subscale content. The questions in Part III investigate the demographic characteristics of the children.

The CEDV scale is globally accepted and has been translated into multiple languages, including Spanish, Urdu, Kurdish and Persian. Particularly, the scale is used in cross-cultural research to address the need for accurate assessment and understanding of the individual’s cultural, linguistic and ethnic background (10).

Existing studies have reported that the CEDV scale is reliable among diverse populations internationally with relatively high overall Cronbach’s coefficients, ranging from 0.79 to 0.97 (11, 12). Similarly, the translated version of the CEDV scale demonstrated a good level of internal consistency, with α coefficients ranging from 0.74 to 0.89 (13–15). The test-retest reliability has shown that the CEDV scale is consistent over time and has indicated a positive correlation in convergent validity testing with other validated questionnaires, namely, the ‘Things I have seen and heard’ and the ‘Exposure to physical aggression’ questionnaire (13). Moreover, existing studies also demonstrated concurrent validity between CEDV scale and several outcomes related to exposure to domestic violence such as anxiety symptoms, maladjustment, reduced self-esteem and physical health complaints (11, 14, 15).

### Study Design, Sampling and Data Collection

**Figure 1:**
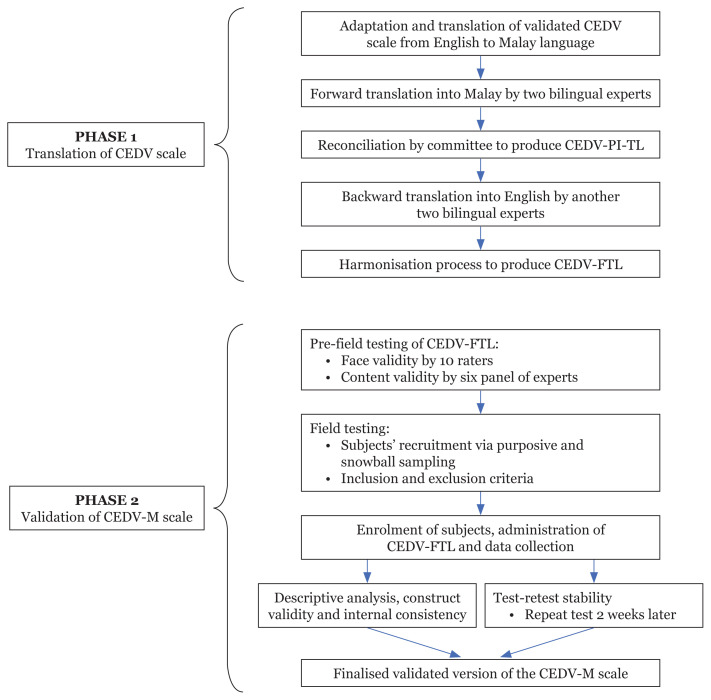
Flowchart of the study

A cross-sectional validation study was conducted in two phases: translation and validation (Figure 1).

#### The Translation Phase

The translation phase involved three important steps: i) forward translation, ii) backward translation and iii) finalisation of the translated version (16). For forward translation, the CEDV scale was translated from the source language, English (CEDV-SL), into Malay, the target language (CEDV-TL). The translators were two bilingual experts, namely, a paediatric medical officer and a Malay language lecturer at the local colleges. Specifically, the experts were knowledgeable about the contents of the scale, as well as the cultural and linguistic nuances in the desired language. The two translations were synthesised into one document (CEDV-PI-TL) after reconciliation among the researchers and translators to address any gaps or differences between the two reports. Thereafter, the CEDV-PI-TL was translated back into English by another two translators, (i.e. a paediatrician and a teacher) during the backward translation process. Furthermore, a committee of six members was formed, comprising the researchers and all the translators involved in the previous process. Both of the backward translated versions were compared against the original version for any ambiguities and discrepancies before the committee members reached a consensus to derive the pre-final version of CEDV (CEDV-FTL). The CEDV-FTL, which is later referred to as the Malay version of the CEDV (CEDV-M), has been used in the study for face validity, content validity and subsequently for psychometric testing.

#### The Validation Phase

##### Content validity

Content validation of the CEDV-M scale was conducted by a panel of six experts who were asked to provide a score of 1 (item not relevant) to 4 (item highly relevant), based on the relevance of the translated items in the CEDV scale. Each face validity index (FVI) was scored as 0 or 1. Subsequently, the content validity index (CVI) was computed by calculating the average scale. Two paediatric medical officers, three general paediatricians and a family medicine specialist assessed the content validity to ensure that the CEDV-M scale is relevant and reflects the original CEDV scale.

##### Face validity

Face validation testing aims to assess the clarity and comprehensibility of the translated items. This assessment was conducted by 10 raters who were children aged 10 years old–16 years old upon obtaining consent from their parents. The children were asked to provide a score for each item in the translated questionnaire using a rating scale of clarity and comprehension which ranged from 1 (item not clear and not understandable) to 4 (item very clear and understandable). Before calculating the FVI, the rating was recorded as 1 (scale 3 or 4) or 0 (scale 1 or 2) and, subsequently computed by calculating the scale average.

##### Validity of the CEDV-M scale

The current study was conducted at a public secondary school in the northern state of Malaysia in Perlis to validate the CEDV-M scale. Before the study, the school authority and the two selected teachers involved in this validation study received a briefing and presentation. The teachers were trained and provided with an approved study protocol to refer to at any time during the study period. The participant information sheet, parental/participant consent and assent forms were provided to the teachers to be distributed to the children. Both teachers and children involved in this study received thorough explanation from the researchers who emphasised confidentially and voluntary participation.

The largest sample needed was to determine the construct validity of the CEDV-M scale using exploratory factor analysis (EFA). By using a sample to variable (42 items in the CEDV-M scale tool) ratio (SVR) of 5:1, a minimum of 231 sample were required, including a 10% drop out rate. Purposive and snowball sampling methods were adopted to include a balanced number for each group of age and gender, as well as to avoid ceiling and floor effects. The recruitment occurred over 2 weeks (16).

The questionnaire was self-administered through an online Google Form platform. The participants received the link to the CEDV-M scale after collecting the consent and assent forms from parents and children who agreed to participate in the study. After 2 weeks, the 30 participants who agreed, were asked to repeat the CEDV-M scale for stability testing. The participants who successfully completed the questionnaire were awarded a certificate of appreciation.

### Data Analysis

Collected data were entered and analysed using the IBM Statistical Packages for Social Sciences (SPSS) version 26.0. Descriptive statistics were used to summarise the socio-demographic characteristics of the participants, where the numerical data were presented as mean (SD) based on their normality distribution, while categorical data presented as frequency (percentage). The raters’ scores were combined and entered into Microsoft Excel to determine content and face validity. However, the indices were calculated manually. The acceptable cut-off score for both the FVI and CVI was 0.80 (17, 18).

The psychometric test used to evaluate the reliability of the questionnaire was Cronbach’s alpha coefficient for internal consistency and inter-class correlation for test-retest stability. The items in the CEDV scale indicated a high internal consistency if the total alpha value was more than 0.6 (19). Stability testing was conducted using the intra-class correlation coefficients (ICC) which measure the consistency of the scale. The ICC estimates and their 95% confidence interval were calculated based on a single rating, absolute agreement and 2-way mixed model. A value less than 0.50 suggested poor reliability, while values between 0.5 and 0.75, 0.75 and 0.90, and values greater than 0.90 indicate moderate, good and excellent reliability, respectively (20).

Construct validity of the CEDV scale was determined using EFA. The Kaiser-Meyer-Olkin (KMO) test was used to measure sampling adequacy and the CEDV scale was considered adequate if the KMO value exceeds 0.6. Furthermore, Bartlett’s test of sphericity was considered significant if the *P*-value was less than 0.05, indicating that there was sufficient non-zero inter-correlation among the analysed items. Subsequently, principal axis factoring with the promax rotation method was conducted, for factors with eigenvalues greater than 1. Items with a loading factor of more than 0.4 were considered to have an acceptable loading factor (21).

## Results

### The Translation, Content Validation and Face Validation of the CEDV-M Scale

During the translation process, a few items in the original questionnaire were identified that, could not be directly translated to adapt to the Malaysian culture and community. In Part III, questions regarding race and ethnicity (item 38), were replaced with Malaysians’ race and ethnicity. To avoid confusion among the children and to nurture Asian values, the term ‘mother’s partner’, which is extensively used in the questionnaire, was replaced with ‘father’ or ‘stepfather’. Since this study was conducted via an online platform, all committee members involved in the translation decided to revise the instruction from the original English version of the CEDV scale. The original instructions were long and might have confused the participants as these were meant to be read by the interviewer during face-to-face interviews.

Content validation of the CEDV-M scale produced an overall validity index of more than 0.80. The item-level content validity index (I-CVI) for all items was either 0.83 or 1.00, the universal agreement index (S-CVI/UA) was 0.88, and the average index (S-CVI/Ave) was 0.98. Thus, all items in the CEDV-M scale demonstrated a satisfactory level of content validity.

For the face validity of this scale, raters found that all items were clear and easy to understand as reflected by all item-level face validity indices (I-FVI) exceeding 0.8. The scale-level face validity index based on average (S-FVI/Ave) and scale-level face validity index based on universal agreement (S-FVI/UA) were 0.97 and 0.8, respectively.

### Psychometric Testing of the CEDV-M Scale

#### Sample Characteristics

In total, this study recruited 235 children between the ages of 13 years old and 16 years old, with a mean age of 14.8 years old (standard deviation [SD] = 1.0). Table 1 shows the demographic background of the children. Particularly, 66.8% (*n* =157) were female and 89.8% (*n* = 211) were of Malay ethnicity while Chinese and Indian students accounted for 6% (*n* = 14) and 4.3% (*n* = 10), respectively. Almost all of the participants lived in their own homes (98.7%, *n* = 232), while three participants (1.3%) lived in their relatives’ houses. Family composition revealed that almost every child lived with at least one of their biological parents and siblings. In total, 94.5% (*n* =222) of the participants stated that they lived with their mothers, while 91.5% (*n* = 215) and 88.5% (*n* = 208), respectively, expressed that they had fathers and siblings around. A portion of them also lived with extended family members, such as grandparents (17.9%, *n* = 42), step parents (2.5%, *n* = 6), and other relatives such as uncles and aunts (2.5%, *n* = 6).

#### Responses to Items

Most of the children completed the questionnaire successfully, except for 14% (*n* = 33) who answered 41 out of 42 items (> 95%). The missing data were from the last item in the CEDV-M scale, item Q42 (“What is your favourite family activity?”), which is an open-ended questionnaire from the sociodemographic part of the scale.

Upon answering the CEDV-M scale, the children reported their exposure to violence at home and in the community. Responses to each item were reviewed according to the subscales (Appendix). Most of the children, had no experience with any level of home violence except during disagreements among the adults in the family. Among these children, 44.7% (*n* = 105) reported events that occurred occasionally, 5.1% (*n* = 12) often and 0.4% (*n* = 1) almost always. Furthermore, approximately one third (27.7%, *n* = 65) claimed that parents sometimes argued about them. Some of them reported that fathers would sometimes hurt their mother’s feelings (22.6%, *n* = 53) and body (5.1%, *n* = 12), and hurt the mother using an object or a weapon (1.3%, *n* = 3). The participants who had experienced any level of home violence were asked to indicate how they knew about the events that occurred. Table 7 (Appendix) indicates that the participants mainly reported that they heard and witnessed the fights up close. Regarding involvement in home violence, 29.4% (*n* = 69) claimed sometimes, 11.9% (*n* = 28) stated that they were often asked by the father to tell their mother. In contrast, less than 10% of the participants had any involvement in violent events at home.

Regarding the risk factors, 39.6% (*n* = 93) of the children had experienced significant changes in their lifetime, such as the death of someone close, their parent’s divorce and moving to a new school or home. Almost half of them (49.4%, *n* = 113) reported that their mothers were sometimes worried and upset, while 1.7% (*n* = 4) reported that their mothers were almost always worried. Moreover, 12.4% (*n* = 29) and 4.7% (*n* = 11) of participants were worried that their fathers and mother, respectively, would become intoxicated or consume drugs, while 0.9% (*n* = 2) were always worried about both parents.

In the community, several children heard a person hurt someone’s feelings. Particularly, 48.5% (*n* = 114) reported sometimes, 18.7% (*n* = 44) often and 3.8% (*n* = 9) almost always. In addition, 39.1% (*n* = 92) reported that sometimes someone hurt their feeling too. A total of 20.9% (*n* = 49) claimed sometimes seen someone get hurt and 8.4% (*n* = 20) do experience themselves. The majority of them had seen someone hurt or killed in a movie (88.2%, *n* = 208), video or mobile game (68.9%, *n* = 162), while 11% claimed that this was almost always true.

For victimisation, 43%, 8.1% and 3.0% of the children reported that were sometimes, often and almost always hurt by family members, respectively. Furthermore, a total of 5.5% had experienced a physical injury. Moreover, 15 children reported having their private parts touched by family members (2.6%, *n* = 6) and non-family members (3.8%, *n* = 9).

#### Construct Validity

EFA was performed for all 42 items using principal axis factoring and Promax rotation. A KMO value of 0.658 which was verified the sampling adequacy while a significant Bartlett’s test of sphericity (*P*-value < 0.001) signified that inter-correlation among items was adequate for the analysis to take place.

Kaiser’s criterion showed 11 factors with Eigenvalues of ≥ 1, while the scree plot only indicated six factors to extract. Factor determination was also examined using parallel analysis, whereby the indicated five-factor solution was the best fit for the data, accounting for 54% of the variance. The original CEDV scale has six subscales, thus, repeated analyses were performed with factor extraction fixed at 5 and 6. The five-factor solution was deemed most conceptually appropriate. However, seven items were removed (Q16, Q17, Q18, Q19, Q24, Q27 and Q33) since the items loading on the factor did not achieve the cut-off point of 0.4, while item Q16 was deleted because of cross-loading in Factors 1 and 3.

From the EFA, items in the two generated factors revealed correlations with the subscales in the original version of the CEDV. Factor 3 comprised items from Q11 to Q14 which correlated with the subscale ‘Involvement in violence’, referred to as Involvement. Factor 5 was labelled ‘Community’ and comprised items Q23, Q25 and Q26, sharing items with the subscale ‘Community violence’ with a newly added item Q32 (“How often has someone who is not in your family touched your private parts?”). Furthermore, Factor 1 was labelled ‘Physical violence’ comprising a total of 10 items, with factor loading ranging from 0.514 to 1.007. Moreover, Factor 2 was labelled ‘Emotional and psychological violence’ and contained 13 items with factor loadings of 0.409 to 0.741. Finally, Factor 4 was labelled ‘Other victims’ and had five items, with a factor loading ranging from 0.573 to 0.963 (Table 2).

#### Internal Consistency

To assess the reliability of the CEDV-M scale, internal consistency was examined using Cronbach’s alpha coefficient. The initial Cronbach’s alpha coefficient for the overall subscales of the CEDV-M scale was calculated according to the original version. All subscales showed relatively high Cronbach’s alpha ranging α = 0.61 to 0.83. The ‘Victimisation’ subscale was the only subscale with a low-reliability coefficient where α = 0.26. However, a satisfactory value of α = 0.52 was obtained after item deleting Q30. The overall α reliability coefficient for the CEDV-M scale was 0.9 and relatively higher than the reliability coefficient of the original CEDV scale. Table 3 provides a detailed comparison.

The five-factor domain model extracted from the EFA with a total of 36 items was analysed for internal consistency. The total α for the CEDV-M scale with five final constructs was 0.87, showing relatively high reliability. Coefficient α for each of factor ranged from α = 0.58 to 0.92 which indicated acceptable internal consistency as illustrated in Table 4. The α for Factor 5 was improved from 0.57 to 0.58 after item Q25 was deleted.

Other psychometric tests performed to assess the reliability of the CEDV-M scale were test-retest stability. The CEDV-M scale was answered twice by a group of 30 children at 2-week intervals. The intraclass correlation coefficient (ICC) was used to examine each of the items in the CEDV-M scale, based on a 2-way mixed effect model with a single rating and absolute agreement. The ICC values for all items ranged from 0.659 to 1.00 indicating that CEDV-M scale has moderate to excellent stability reliability. Moreover, 58% of the items (25 items) had excellent stability and reliability (Table 5).

## Discussion

The CEDV scale was developed by Edleson et al. in 2008 to address the limitation of available tools to measure children’s experience of domestic violence (8). The CEDV-M scale has undergone substantial translation and adaptation to ensure the integrity of this study instrument. The final construct of the CEDV-M scale consists of 36 items framed within five-factor domains: ‘Physical violence’, ‘Emotional and psychological violence’, ‘Involvement’, ‘Home exposure’ and ‘Community exposure’.

The current study used an online platform that had led to few advantages over usual interview method. For example, online form addressed issues with missing data as it was compulsory to answer all critical questions before submitting the CEDV-M scale form. Furthermore, the response rate was high (100%), most likely because the flexible times allowed the children to complete the form anonymously. The researchers ensured that the instructions were simple but concise for the children to understand and answer all the questions successfully. The high response rate and minimal missing data were also attributed to the readability, clarity and comprehensibility of the developed questionnaire. This was reflected in the satisfactory FVI. Moreover, the developed CEDV-M scale proved to be relevant and had an appropriate degree of representativeness to its targeted construct based on an acceptable CVI.

Regarding construct validity, EFA produced five factors for the CEDV-M scale as opposed to six as in the original version. The final construct also conceptually examines type of violence. For all five factors domains extracted, the respective items loaded with > 0.4 factor loading exhibited acceptable communalities without cross-loading. Furthermore, two new factors or subscales were found, namely, ‘Physical violence’ and ‘Emotional and psychological violence’. ‘Physical violence’ comprised of 10 items that explained 30.6% of the variance, with high factor loading (0.514–1.007) and good internal consistency (α = 0.854). ‘Emotional and psychological violence’ comprised of total 13 items, with acceptable factor loadings ranging from 0.409 to 0.741 and good internal consistency (α = 0.839). The other three factor domains with their respective items exhibited some similarities with the original version. Moreover, the factor loading and communalities were acceptable with relatively good internal consistency, except for ‘Community violence’ which comprised the least number of items (i.e. three items after Q25 was deleted to improve the correlation coefficient). This factor had borderline α = 0.58, which most likely contributed to the small number of items and the homogenous sample of the participants. However, the total Cronbach’s alpha value was relatively high.

Several factors contributed to the variations in alpha between the original and the Malay translations. Particularly, the variations resulted from the fewer items on the ‘Factor community exposure’ and the need for careful cultural adaptation during the translation process. Q25 asked participants “How frequently do you intentionally damage a person physically, such as by hitting, kicking, or other similar actions?” While adaptation may not be a common action in the context of the current population, this may differ in children with Western values. The alpha of 0.58 in the ‘Factor community exposure’ domain could be considered acceptable (18). Even though several qualitative descriptors have been used to interpret alpha values in the literature, no clear consensus has been reached regarding the acceptable alpha values calculated (18). Another potential reason may be the sampling issue which could not capture the positive response in that particular domain.

During the original development and validation study of the CEDV scale, EFA was attempted, but it failed to generate coherent factors because of the small sample size. Therefore, subscales of the original version were not tested. Two previous studies conducted an EFA on the translated CEDV scale yielded in seven and five factor domains respectively; however, no details about the analysis were provided for comparison. The CEDV-M scale has been proven to be stable and reliable over time. All the items in CEDV-M scale had acceptable ICC values, with more than 90% demonstrating good to excellent ICC values > 0.75, indicating stability and reliability.

## Conclusion

The current study found that the CEDV-M scale can facilitate researchers and authorities in Malaysia to measure a child’s experience with domestic violence from their perspective. The scale has excellent validity, satisfactory reliability and good stability over time. However, the CEDV-M scale measures a slightly different dimension from the original version which is required to perform further confirmatory factor analysis (CFA) and standard setting for the scoring system.

## Limitations

This study has several limitations. First, CEDV-M scale was translated and adapted to the Malay language. Consequently, it cannot be utilised by children who are not fluent in reading and understanding the formal Malay language. Therefore, further studies to translate and validate the scale to other languages, such as Mandarin and Tamil, would increase the accessibility of its use among the multiracial population in Malaysia. Second, even though snowball sampling was already incorporated within the sampling method, the method may still contribute to the homogeneity of the responses based on the single-centre study area. Thus, to improve response distribution, subsequent research ought to recruit a larger number of samples and include individuals with increased likelihood of exposure to domestic violence, such as children living in welfare shelters, which is particularly important in prevalence studies.

As previously discussed, it is important to determine the construct validity of the CEDV scale as available data supporting the current subscales in the original CEDV scale remains limited. Thus, this study recommends that subsequent research re-evaluate using EFA while proceeding with CFA to further evaluate the validity of the construct. Moreover, the standard setting of the scoring system is important to explore in future studies.

## Figures and Tables

**Table 1:** Characteristics of the participants (*N* = 235)

Variable	Mean (SD)	*n*	%
Age (years old)	14.8 (1.0)		
13		22	9.4
14		70	29.8
15		59	25.1
16		84	35.7
Gender			
Male		78	33.2
Female		157	66.8
Ethnic			
Malay		211	89.8
Chinese		14	6.0
Indian		10	4.3
Where the child lived			
Own house		232	98.7
Relative’s house		3	1.3
Shelter		0	0
People child lived with (multiple answer possible)			
Mother		222	94.5
Father		215	91.5
Grandmother		27	11.5
Grandfather		15	6.4
Siblings		208	88.5
Step-parents		6	2.5
Others		6	2.5

**Table 2:** Items, factor loadings and domains of the CEDV-M scale

Item no.	Item	Rotated factor loadings				

		1 Physical violence	2 Psychological violence	3 Involvement	4 Other victims	5 Community violence
Q9	Father threatened to use weapon/object	1.007				
Q8-1	Father hurt mother’s body	0.988				
Q9-1	Father threatened to use weapon/object	0.965				
Q10	Father hurt mother with weapon/object	0.891				
Q10-1	Father hurt mother with weapon/object	0.887				
Q8	Father hurt mother’s body	0.710				
Q4-1	Father stopped mother from eating/sleeping	0.698				
Q15	Father did something to you to scare mother	0.573				
Q4	Father stopped mother from eating/sleeping	0.516				
Q3-1	Father stopped mother from doing something	0.514				
Q28	Seen someone hurt or killed in movie		0.741			
Q5-1	Parents argued about you		0.720			
Q5	Parents argued about you		0.698			
Q29	Seen someone hurt or killed in video/mobile game		0.668			
Q1	Adult in family disagree		0.663			
Q30	Adult in family hurt your feeling		0.662			
Q1-1	Adult in family disagree		0.658			
Q22	Heard person hurt other’s feeling		0.550			
Q20	Mother seem sad, worried or upset		0.549			
Q2	Father hurt mother’s feelings		0.518			
Q3	Father stopped mother from doing something		0.449			
Q21	Had big changes in life		0.430			
Q2-1	Father hurt mother’s feelings		0.430			
Q13	Called for help			0.976		
Q11	Yelled at parents during fight (different room)			0.891		
Q12	Yelled at parents during fight (same room)			0.831		
Q14	Physically tried to stop fight			0.783		
Q6	Father hurt pet				0.963	
Q6-1	Father hurt pet				0.931	
Q31	Adult in family hurt your body				0.621	
Q7-1	Father destroyed things				0.598	
Q7	Father destroyed things				0.516	
Q32	Someone not in family touched your private parts					0.564
Q23	Someone hurt your feeling					0.549
Q26	Seen someone get hurt					0.485
Q25	You physically hurt a person					0.480

	Eigenvalues	12.478	4.140	3.364	2.420	2.351
	% of variance	29.71	9.851	8.010	5.763	5.598

**Table 3:** Internal consistency reliability of CEDV-M scale and original version CEDV scale according to subscales

Subscale	Total *n* of items	Alpha	

		CEDV-M scale	CEDV scale
Home violence	10	0.83	0.74
Home exposure	10	0.80	0.76
Involvement	7	0.81	0.50
Community exposure	8	0.76	0.71
Risk factors	4	0.61	0.60
Victimisation	4	0.52 (after item deleted)	0.70

Total		0.91	0.84

**Table 4:** Internal consistency reliability of CEDV-M with new subscales

Factor	*N* of item	α	α (after items deleted)
Physical violence	10	0.854	
Emotional and psychological violence	13	0.839	
Involvement	4	0.927	
Other victims	5	0.681	
Community violence	4	0.57	0.58

Total	36	0.87	

Note: Cronbach alpha

**Table 5:** Intraclass correlation coefficient of all items in CEDV-M scale (*n* = 30)

Items	ICC (3, 1)	95% CI	

		Lower	Upper
Level of home violence			
Q1	0.865	0.738	0.933
Q2	0.880	0.764	0.941
Q3	0.873	0.753	0.937
Q4	0.873	0.753	0.937
Q5	0.861	0.726	0.932
Q6	1.00	–	-
Q7	0.937	0.827	0.969
Q8	0.90	0.802	0.957
Q9	1.00	–	-
Q10	0.659	0.40	0.821
Home exposure			
Q1-1	0.938	0.963	0.992
Q2-1	0.978	0.955	0.990
Q3-1	0.980	0.959	0.990
Q4-1	0.982	0.991	106
Q5-1	0.990	0.979	0.995
Q6-1	0.976	0.950	0.988
Q7-1	0.994	0.988	0.997
Q8-1	0.992	0.984	0.996
Q9-1	0.982	0.961	0.991
Q10-1	1.00	–	–
Involvement			
Q11	1.00	–	–
Q12	0.721	0.496	0.856
Q13	0.885	0.773	0.944
Q14	0.912	0.825	0.957
Q15	0.721	0.496	0.856
Q16	0.868	0.741	0.935
Q17	0.689	0.743	0.935
Risk factors			
Q18	0.919	0.838	0.961
Q19	1.00	–	–
Q20	0.886	0.765	0.946
Q21	0.961	0.921	0.981
Community exposure			
Q22	0.889	0.782	0.945
Q23	0.882	0.757	0.943
Q24	0.819	0.653	0.910
Q25	0.788	0.604	0.893
Q26	0.873	0.751	0.937
Q27	0.940	0.841	0.961
Q28	0.827	0.644	0.917
Q29	0.941	0.879	0.971
Victimisation			
Q30	0.873	0.752	0.938
Q31	1.00	–	–
Q32	1.00	–	–
Q33	1.00	–	–

Notes: ICC = intraclass correlation coefficient; CI = confidence interval

## Appendix

**Table 6:** Frequency and percentage of items in six subscales (*N* = 235)

Item	Never *n* (%)	Sometimes *n* (%)	Often *n* (%)	Almost always *n* (%)
Level of home violence				
Q1. Adult in family disagree	117 (49.8)	105 (44.7)	12 (5.1)	1 (0.4)
Q2. Father hurt mother’s feelings	178 (75.7)	53 (22.6)	2 (0.9)	2 (0.9)
Q3. Father stopped mother from doing something	213 (90.6)	22 (9.4)	0 (0)	0 (0)
Q4. Father stopped mother from eat or sleep	223 (94.9)	12 (5.1)	0 (0)	0 (0)
Q5. Parents argued about you	165 (70.2)	65 (27.7)	4 (1.7)	1 (0.4)
Q6. Father hurt pet	226 (96.2)	9 (3.8)	0 (0)	0 (0)
Q7. Father destroyed things	211 (89.8)	22 (9.4)	2 (0.9)	0 (0)
Q8. Father hurt mother’s body	222 (94.5)	12 (5.1)	1(0.4)	0 (0)
Q9. Father threatened to use weapon/object	228 (97.0)	7 (3.0)	0 (0)	0 (0)
Q10. Father hurt mother using weapon/object	232 (98.7)	3 (1.3)	0 (0)	0 (0)
Involvement in violent event				
Q11. Yelled at parents during fight (different room)	225 (95.7)	8 (3.4)	0 (0)	2 (0.9)
Q12. Yelled at parents during fight (same room)	219 (93.2)	14 (6)	0 (0)	2 (0.9)
Q13. Called for help	224 (95.3)	5 (2.1)	2 (0.9)	4 (1.7)
Q14. Physically tried to stop fight	219 (93.2)	13 (5.5)	1 (0.4)	2 (0.9)
Q15. Father did something to you to scare mother	223 (94.9)	12 (5.1)	0 (0)	0 (0)
Q16. Tried to get away from fighting	215 (91.5)	17 (7.2)	3 (1.3)	0 (0)
Q17. Father asked to tell on mother	137 (58.3)	69 (29.4)	28 (11.9)	1 (0.4)
Risk factors				
Q18. Worry about father’s drinking or taking drugs	206 (87.7)	19 (8.1)	8 (3.4)	2 (0.9)
Q19. Worry about mother’s drinking or taking drugs	224 (95.3)	9 (3.8)	0 (0)	2 (0.9)
Q20. Mother seem sad, worried or upset	97 (41.3)	116 (49.4)	18 (7.7)	4 (1.7)
Q21. Had big changes in life	142 (60.4)	79 (33.6)	14 (6.0)	0 (0)
Community exposure				
Q22. Heard person hurt other’s feeling	68 (28.9)	114 (48.5)	44 (18.7)	9 (3.8)
Q23. Someone hurt your feeling	133 (56.6)	92 (39.1)	9 (3.8)	1 (0.4)
Q24. You hurt other’s feeling	184 (78.3)	49 (20.9)	2 (0.9)	0 (0)
Q25. You physically hurt a person	227 (96.6)	8 (3.4)	0 (0)	0 (0)
Q26. Seen someone get hurt	182 (77.4)	49 (20.9)	4 (1.7)	0 (0)
Q27. Someone hurt you	215 (91.5)	19 (8.0)	1 (0.4)	0(0)
Q28. Seen someone hurt or killed in movie	27 (11.5)	108 (46.0)	73 (31.1)	27 (11.5)
Q29. Seen someone hurt or killed in video/mobile game	73 (31.1)	77 (32.8)	59 (25.1)	26 (11.1)
Victimisation				
Q30. Adult in family hurt your feeling	108 (46)	101 (43)	19 (8.1)	7 (3.0)
Q31. Adult in family hurt your body	222 (94.5)	12 (5.1)	1 (0.4)	0 (0)
Q32. Someone in family touched your private parts	226 (96.2)	9 (3.8)	0 (0)	0 (0)
Q33. Someone not in family touched your private parts	229 (97.4)	6 (2.6)	0 (0)	0 (0)

**Table 7:** Frequency of the level of exposure to home violence

Item	*n*	Saw the outcome	Heard afterwards	Heard while it was happening	Saw it from far while it was happening	Saw it and was near while it was happening
Home exposure						
Q1-1. Adult in family disagree	118	0	17	45	25	30
Q2-1. Father hurt mother’s feelings	59	0	10	20	6	26
Q3-1. Father stopped mother from doing something	22	0	9	7	5	6
Q4-1. Father stopped mother from eat or sleep	12	1	3	3	1	3
Q5-1. Parents argued about you	70	0	6	19	7	30
Q6-1. Father hurt pet	9	4	1	0	3	1
Q7-1. Father destroyed things	24	4	1	4	9	7
Q8-1. Father hurt mother’s body	13	2	3	2	3	4
Q9-1. Father threatened to use weapon/object	7	0	0	0	4	3
Q10-1. Father hurt mother using weapon/object	3	0	0	0	1	2

**Table 8:** Face validity index

Item	Rater 1	Rater 2	Rater 3	Rater 4	Rater 5	Rater 6	Rater 7	Rater 8	Rater 9	Rater 10	Experts in Agreement	l-FVl	UA
Q1	1	1	1	1	1	1	1	1	1	1	10	1	1
Q2	1	1	1	1	1	1	1	1	1	1	10	1	1
Q3	0	1	1	1	1	1	1	1	1	1	9	0.9	0
Q4	0	1	1	1	1	1	1	1	1	1	9	0.9	0
Q5	1	1	1	1	1	1	1	1	1	1	10	1	1
Q6	1	1	1	1	1	1	1	1	1	1	10	1	1
Q7	1	1	1	1	1	1	1	1	1	1	10	1	1
Q8	1	1	1	1	1	1	1	1	1	1	10	1	1
Q9	1	1	1	1	1	1	1	1	1	1	10	1	1
Q10	1	1	1	1	1	1	1	1	1	1	10	1	1
Q11	0	1	1	1	1	1	1	1	1	0	8	0.8	0
Q12	0	1	1	1	0	1	1	1	1	1	8	0.8	0
Q13	1	1	1	1	1	1	1	1	1	1	10	1	1
Q14	1	1	1	1	1	1	1	1	1	1	10	1	1
Q15	1	1	1	1	1	1	1	1	1	1	10	1	1
Q16	1	1	1	1	1	1	1	1	1	1	10	1	1
Q17	0	1	1	1	1	1	0	1	1	1	8	0.8	0
Q18	1	1	1	1	1	1	1	1	1	1	10	1	1
Q19	1	1	1	1	1	1	1	1	1	1	10	1	1
Q20	1	1	1	1	1	1	1	1	1	1	10	1	1
Q21	1	1	1	1	1	1	1	1	1	1	10	1	1
Q22	1	1	1	1	1	1	1	1	1	1	10	1	1
Q23	1	1	1	1	1	1	1	1	1	1	10	1	1
Q24	1	1	1	1	1	1	1	1	1	1	10	1	1
Q25	1	1	1	1	1	1	1	1	1	1	10	1	1
Q26	1	1	1	1	1	1	1	1	1	1	10	1	1
Q27	0	1	1	1	1	1	1	1	1	1	9	0.9	0
Q28	1	1	1	1	1	1	1	1	1	1	10	1	1
Q29	1	1	1	1	1	1	1	1	1	1	10	1	1
Q30	1	1	1	1	1	1	1	1	1	1	10	1	1
Q31	1	1	1	1	1	1	1	1	1	1	10	1	1
Q32	1	1	1	1	1	1	1	1	1	1	10	1	1
Q33	1	1	1	1	1	1	1	1	1	1	10	1	1
Q34	1	0	1	1	0	1	1	1	1	1	8	0.8	0
Q35	1	1	1	1	1	1	1	1	1	1	10	1	1
Q36	1	1	1	1	1	1	1	1	1	1	10	1	1
Q37	1	1	1	1	1	1	1	1	1	1	10	1	1
Q38	1	1	1	1	1	1	1	1	1	1	10	1	1
Q39	1	1	1	1	1	1	1	1	1	1	10	1	1
Q40	1	0	1	1	1	1	1	1	1	1	9	0.9	0
Q41	1	1	1	1	1	1	1	1	1	1	10	1	1
Q42	1	1	1	1	1	1	1	1	1	1	10	1	1
												
											s-FVl/Ave	0.97	
Proportion relevance	0.86	0.98	1	1	0.95	1	0.98	1	1	0.98	S-FVI/UA		0.8
		
Average proportion of items judged as relevance across the experts											0.97		

**Table 9:** Content validity index

Item	Expert 1	Expert 2	Expert 3	Expert 4	Expert 5	Expert 6	Experts in Agreement	I-CVI	UA
Q1	1	1	1	1	1	1	6	1	1
Q2	1	1	0	1	1	1	5	0.83	0
Q3	1	1	1	1	1	1	6	1	1
Q4	1	1	1	1	1	1	6	1	1
Q5	1	1	1	1	1	1	6	1	1
Q6	1	1	1	1	1	1	6	1	1
Q7	1	1	1	1	1	1	6	1	1
Q8	1	1	1	1	1	1	6	1	1
Q9	1	1	1	1	1	1	6	1	1
Q10	1	1	1	1	1	1	6	1	1
Q11	1	1	1	1	1	1	6	1	1
Q12	1	1	1	1	1	1	6	1	1
Q13	1	1	1	1	1	1	6	1	1
Q14	1	1	1	1	1	1	6	1	1
Q15	1	1	1	1	1	1	6	1	1
Q16	1	1	1	1	1	1	6	1	1
Q17	1	1	1	1	1	1	6	1	1
Q18	1	1	1	1	1	1	6	1	1
Q19	1	1	1	1	1	1	6	1	1
Q20	1	1	1	1	1	1	6	1	1
Q21	1	0	1	1	1	1	5	0.83	0
Q35	1	1	1	1	1	1	6	1	1
Q34	1	1	1	1	1	1	6	1	1
Q22	1	1	1	1	1	1	6	1	1
Q23	1	1	1	1	1	1	6	1	1
Q24	1	1	1	1	1	1	6	1	1
Q25	1	1	1	1	1	1	6	1	1
Q26	1	1	1	1	1	1	6	1	1
Q27	1	1	1	1	1	1	6	1	1
Q28	1	1	0	1	1	1	5	0.83	0
Q29	1	1	0	1	1	1	5	0.83	0
Q30	1	1	1	1	1	1	6	1	1
Q31	1	1	1	1	1	1	6	1	1
Q32	1	1	1	1	1	1	6	1	1
Q33	1	1	1	1	1	1	6	1	1
								
							S-CVl/Ave	0.98	
Proportion relevance	1	0.97	0.91	1	1	1	S-CVI/UA		0.88
		
Average proportion of items judged as relevance across the experts							0.98		
